# Prevalence of *Microsporum canis* from Pet Cats in Small Animal Hospitals, Chiang Mai, Thailand

**DOI:** 10.3390/vetsci9010021

**Published:** 2022-01-09

**Authors:** Vena Chupia, Jirapat Ninsuwon, Kakanang Piyarungsri, Chollada Sodarat, Worapat Prachasilchai, Witaya Suriyasathaporn, Surachai Pikulkaew

**Affiliations:** 1 Department of Veterinary Biosciences and Public Health, Faculty of Veterinary Medicine, Chiang Mai University, Chiang Mai 50100, Thailand; vena.ch@cmu.ac.th; 2 Research Center of Producing and Development of Products and Innovations for Animal Health and Production, Faculty of Veterinary Medicine, Chiang Mai University, Chiang Mai 50100, Thailand; witaya.s@cmu.ac.th; 3 Veterinary Diagnostic Center (VDC), Chiang Mai University Animal Hospital, Faculty of Veterinary Medicine, Chiang Mai University, Chiang Mai 50100, Thailand; faiyjirapat@gmail.com; 4 Department of Companion Animals, Faculty of Veterinary Medicine, Chiang Mai University, Chiang Mai 50100, Thailand; kakanang.p@cmu.ac.th (K.P.); chollada.s@cmu.ac.th (C.S.); worapat.p@cmu.ac.th (W.P.); 5 Department of Food Animal Clinics, Faculty of Veterinary Medicine, Chiang Mai University, Chiang Mai 50100, Thailand

**Keywords:** cat, dermatophytes, *Microsporum canis*, zoonosis, prevalence, Chiang Mai

## Abstract

Dermatophytosis is a disease caused by dermatophytes, a group of fungi that can cause disease both in humans and animals. The important genera that are pathogenic in animals include *Trichophyton* and *Microsporum. Microsporum canis* is an important species because it can cause zoonosis and is commonly found in domestic animals. Cats, which live very close to humans, may expose humans to this pathogen. This research focused on the epidemiology of *M. canis* found in cats. Hair samples were collected via the Mackenzie technique from cats with and without skin lesions, preliminarily examined with 10% KOH preparation, and cultured for fungal identification. Samples were confirmed with molecular techniques including polymerase chain reaction, gel electrophoresis, and sequencing. Samples were collected from 138 cats located in 93 households, 43 from cats with skin lesions (31.16%) and 95 from cats without skin lesions (68.84%). Eighteen cats with lesions (13.04%) and ten cats without lesions (7.2%) were found to carry *M. canis*. In eleven of the eighteen cats both with skin lesions and positive for *M. canis* (61.11%), the pathogen was found both at the site of the lesion and at other sites in the body. Because the pathogen can be found in the hair of cats with and without skin lesions, owners, keepers, veterinarians, and others who come into contact with these animals are at risk of infection if they are not aware or do not take precautions after contact with them.

## 1. Introduction

Dermatophytosis, or ringworm, can cause disease in both humans and animals. The lesions occur on the skin, especially the skin, as well as the hair and nails. Dermatophytosis is caused by fungi belonging to the dermatophytes group, which has more than 40 species across the following seven genera: *Trichophyton, Epidermophyton, Nannizzia, Lophophyton, Paraphyton, Microsporum, and Arthroderma.* [1,2]. The genera *Trichophyton* and *Microsporum* are the main causes of ringworm in dogs and cats, including *Microsporum canis*, *Nannizzia gypsea,* and *Trichophyton mentagrophytes.* Infected cats can transmit diseases to other cats and cause ringworm in humans. Today, the number of patients with fungal skin diseases, especially dermatophytosis or ringworm, has increased. In 2005–2010, 20–25% of the world’s civilians [3], especially those living in tropical, hot, and humid regions, were infected with dermatophytosis. These pathogenic fungi flourish well at an estimated temperature of 25–28 °C. Large and crowded populations cause easy exposure to the infection. Pet animals, which can easily cause fungal infections to be passed from animals to people. This causes larger numbers of cases of eczema skin diseases in the tropics than in other regions.

In general, cats are absolutely the main reservoir for *M. canis*, and if the disease occurred, the infected cat may have symptoms such as hair loss, blisters, papules, scabs, dandruff, scales and crust, erythema, follicular plugging, hyperpigmentation, abnormal nail growth, and pruritus. The cat may scratch, and this causes subsequent skin trauma, followed by purulent dermatitis (pyotraumatic dermatitis) or ulcerative eosinophilic lesions [4]. The lesions are usually found on the face, ears, nose, paws, and other body parts [5]. However, dermatophytes can also be found in healthy cats that do not show any symptoms or lesions. One study found that fifteen genera of fungi were detected from the skin of healthy cat, thirteen genera were contaminant fungi which can found in the environment, but only two genera were dermatophytes, which were Microsporum and Trichophytons [6]. Another study found that 584 of 5644 cats (10.3%) were positive for dermatophytes, and 381 cats (6.75%) had dermatitis. However, only 94 both had skin lesions and gave a positive result in fungal culture, with about 490 others (8.68%) having fungi detected on the skin but showing no signs or symptoms of skin disease [7]. The most common dermatophyte in cats is *M*. *canis*.

Pathogen transmission depends on many factors, especially spore contraction through direct contact with a carrier cat, which is the main factor that causes the spread of the disease. In addition, contact with contaminated spores in blankets or mattress cushions, grooming, gloves, or even external parasites that are present on infected cats can be the cause of transmission [8]. In addition to contact from cat to cat, cat owners, cat breeders, veterinarians and assistants who care for cats may have a higher risk of fungal infections and subsequent eczema. This research showed that healthy cats without skin lesions may carry dermatophytes on their bodies [7], which can be transmitted to other animals or humans.

## 2. Materials and Methods

### 2.1. Sample Collection

Hair samples from 95 healthy cats without skin lesions and 43 cats with skin lesions were collected by the technique of brushing with a toothbrush (Mackenzie technique) [9] after taking a general history and health record. For cats with skin lesion, two samples were taken as follows: “lesion”, a sample from the side of the lesion area; “other site of body”, a sample from other areas of the body always in the cat with lesion. For cat without lesion, one sample was taken as follows: “body”, a sample random by brushing the hair on not less than 1/3 of the body.” Samples were kept sealed in sterile plastic bags at room temperature. Hair samples were then subjected to preliminary determination by 10% KOH preparation and the samples were cultured within 24 h [10].

### 2.2. Fungal Identification

Sterilized artery forceps were used to pull hairs from the brush. Hairs were placed on the surface of Sabouraud’s dextrose agar and Mycosel agar, and then incubated at 25 °C for 10–14 days [11].

Macroscopic morphology features recorded were the size, texture and color of colonies. The microscopic examination was performed by staining the fungus with lactophenol cotton blue, and then examining the important characteristics of the fungus under 100× and 400× microscope magnification to observe the fungal characteristics, namely, the size, shape, and arrangement of microconidia and macroconidia [11].

### 2.3. Confirmation of Species Using Molecular Techniques

To confirm of fungal species, polymerase chain reaction (PCR) and sequencing were applied from 3 samples of fungal culture. The DNA of some isolates suspected to be *M. canis* was extracted using a NucleoSpin PlantII kit^®^ (Machery-Nagel GmbH, Dauren, Germany). DNA concentration and purity were determined by spectrophotometer (Du 730, Beckman Coulter^®^, Brea, CA, USA), and the samples were stored at −80 °C until further analysis. 

PCR was performed using ITS1 (TCC GTA GGT GAA CCT GCG G) and ITS4 (TCC GCT TAT TGA TAT GC) as primers for the region in the 16sRNA gene (according to White et al. 1990 [12]). The 25 µL PCR reaction volume contained the following: 5µM of each primer, 2× QuickTaq DyeMix^®^ (TOYOBO, Osaka, Japan), and DNA template. The PCR conditions were as follows: 94 °C (2 min); 40 cycles of 94 °C (30 s), 58 °C (30 s), and 72 °C (30 s); final extension at 72 °C (7 min). The amplicon size was 715 base pairs. The molecular size of amplicons was determined by electrophoresis (in 1.5% agarose gel in Tris-borate-ethylene diamine tetra-acetic acid (TBE) gel in 0.5 TBE buffer stained with RedSafe^®^ (iNtRON Biotechnology Inc., Gyeonggi-do, Korea). The DNA bands images were captured using a 148 Gelmax™ Imager (Ultra-Violet Products, Cambridge, UK). The nucleotide sequences of the amplicons were determined and compared to the sequences in the GeneBank database.

### 2.4. Data Analysis

The prevalence of *M. canis* contamination in cats with and without lesions was calculated using descriptive statistics and a confidence interval (CI) of 95%. 

## 3. Results

### 3.1. Sample Collection

**Figure 1:**
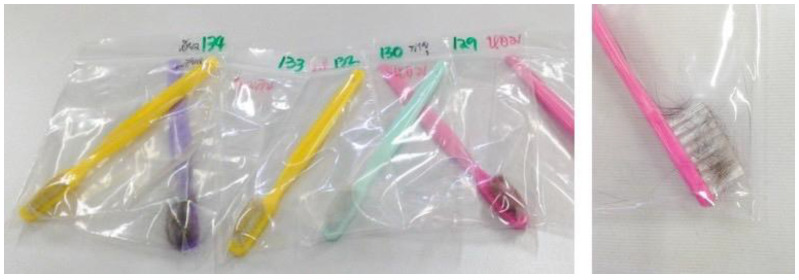
Some of the samples collected from cats by the Mackenzie.

A total of 175 samples of cat hair were collected using the Mackenzie technique (Figure 1) from 138 cats from 93 different owners, with most samples coming from animals registered at the small animal hospital of the Faculty of Veterinary Medicine, Chiang Mai University (46.38%). Other cats (53.62%) were located through animal clinics in Chiang Mai. Of the total 138 cats, 43 had skin lesions (31.16%) and 95 did not have lesions (68.84%). *M. canis* was detected in eighteen out of 43 cats with skin lesions (41.86%; CI = 27.16–56.61), and in ten out of 95 cats without lesions (10.53%). 

### 3.2. Fungal Identification

**Figure 2:**
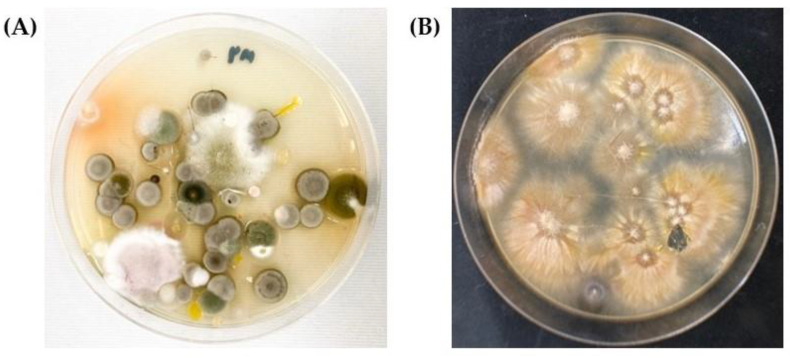
Culture isolation, (**A**) Fungal colonies from the sample that flourish on Sabouraud’s dextrose agar, incubation at 25 °C, 7–10 days (**B**) Fungal colonies of *M. canis* on Sabouraud’s dextrose agar, incubation at 25 °C, 14 days.

**Figure 3:**
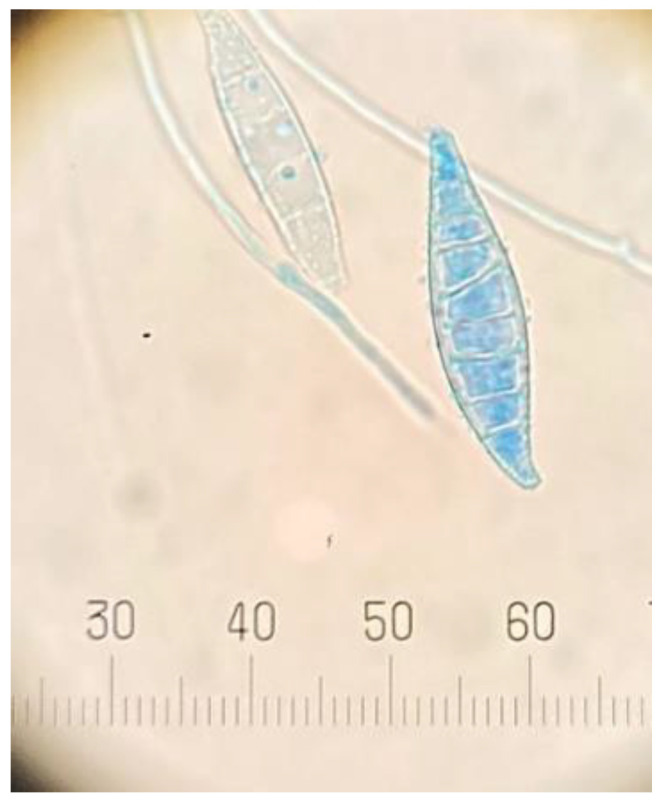
Macroconidia of *M. canis* stained with lactophenol cotton blue under microscope (400× magnification).

All of the samples were tested with a 10% KOH preparation, which did not show the presence of any mold, arthroconidia, or fungal spores. The samples were then cultured and diagnosed (Figure 2 and Figure 3).

Based on a total of 138 cats, 28 (20.29%; CI = 13.58–27.0) were positive for *M. canis*. Eighteen of the positive results (64.29%; CI = 46.53–82.03) were found in cats with skin lesions; in this group, *M. canis* was found in samples from both the lesion and other sites in nine cases (50%; CI = 26.90–73.10), in samples from the non-lesion sites only in two cases (11.11%; CI = 3.41–25.63), and in samples from lesion sites only in seven cases (38.89%; CI = 16.37–61.41). Of the 28 cats that were positive for *M. canis*, ten did not have skin lesions (35.71%; CI = 17.97–53.46) (Table 1). Some of the samples were confirmed by PCR.

### 3.3. Confirmation of Species Using Molecular Techniques

**Figure 4:**
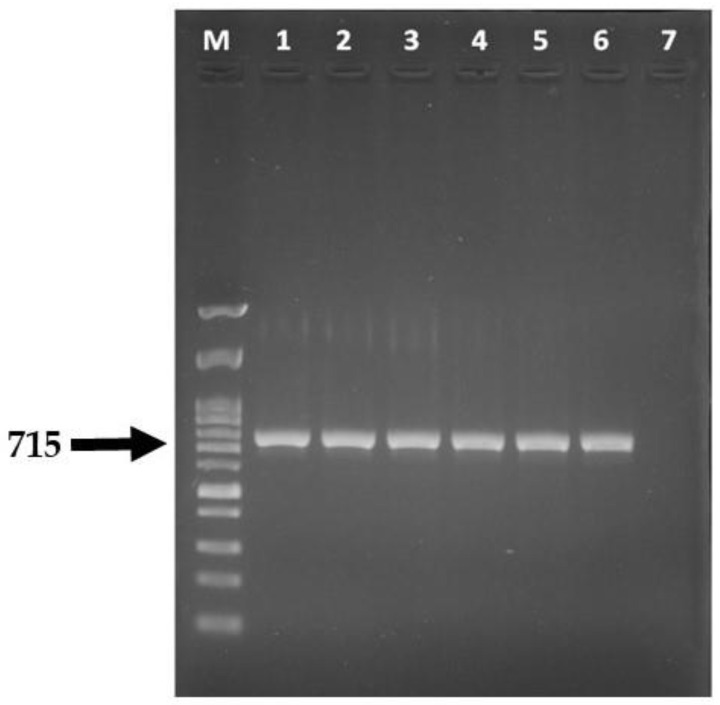
Agarose gel showing amplification of the part of the small subunit of ribosomal RNA gene using primers ITS1 and ITS4. Lane M = DNA marker 100 bp ladder, Lanes 1–5 = PCR product of sample for which *M. canis* was suspected, Lane 6 = PCR product of fungal positive control and Lane 7 = no amplification in negative control.

Fungi suspected to be *M. canis* (3 isolates) were confirmed by PCR using primers specific for a partial gene in the small subunit of the ribosomal RNA gene (18S rRNA gene). The PCR product was about 715 base pairs (Figure 4). Sequencing data were analyzed using the BLAST program under the NCBI database. The analysis found that all sequences were *M. canis.*

## 4. Discussion

An epidemiological study by the World Health Organization reported that there are three groups of dermatophytes that can cause infections in both humans and animals. These three groups, divided by the habitat and preferences of the fungus, are (1) anthropophilic dermatophytes, (2) zoophilic dermatophytes, and (3) geophilic dermatophytes [1,2]. Anthropophilic dermatophytes usually cause disease in humans; however, some species in this group can cause disease in animals, since they grow well in keratinized tissue. This allows the infection to spread from person to person via infected scabs or skin fragments. This group includes *T. rubrum*, *M. audouinii*, *Epidermophyton floccosum* (*E. floccosum*), *T. tonsurans*, and *T. megnini*. The fungi in the zoophilic dermatophyte group usually cause disease in animals but can also infect humans and cause ringworm. This group includes *M. canis*, *M. equinum*, *M. gallinae*, *T. verrucosum*, and *T. mentagrophytes*. The last group is geophilic dermatophytes, which thrive in soil, plants, and environment habitats. Humans or animals can become infected by this group of fungi through contact with the environment or soils, or they may be exposed via abrasions or wounds to the skin. The members of this group include *N. gypsea*, *M. nanum*, *M. persicolor*, and *M. cookei.* Some studies have found that dermatophytes are dispersed across different regions of the world, with the behavior of the population in each area depending on the climate, humidity, and residential characteristics [2,3]. *Trichophyton rubrum*, *T. mentagrophytes*, *T. verrucosum*, *T. violaceum*, *T. tonsurans*, *M. canis*, *N. gypsea*, *M. audouinii*, and *E. floccosum* are the most common pathogens in Asian countries [1,2].

Most fungal skin diseases suffered by cats are caused by *M. canis* [13,14], which belongs in the group of zoophilic dermatophytes. The natural habitat of this fungus is in animals; however, this infection can also cause ringworm in humans in different parts of the body, such as the nails [15] and head [16]. Therefore, this research aimed to detect this pathogen in cats, which is a pet species that lives very close to humans and may expose humans to diseases.

According to this research, the prevalence of *M. canis* in cats with and without skin lesions in Chiang Mai is 20.29%. Previous studies have also looked at this group of cats that do not show skin lesions. In addition, the prevalence of the infection was determined in both short-haired and long-haired cats (6.25% and 34.92%, respectively) [17], and *M. canis* was observed in normal rabbits in rabbit cafes, and it was found in both normal rabbits and rabbits with skin lesions [18]. Previous studies have shown that *M. canis* can be found in normal, healthy animals; there may not be skin lesions, which is a risk factor for humans being exposed without knowing. Humans or animals in a state of weakness, illness, or low/impaired immunity can suffer from this fungal disease. Therefore, if quick diagnostic methods are developed for faster diagnosis and treatment, the chance of humans and animals suffering from the disease is likely to be reduced.

In this study, 20.29% of cat was positive for *M. canis*; 13.04% was cat with skin lesion and 7.25% was cat without skin lesion. Similarly, Verbrugge et al. [7] found that 8.68% of cats without skin disease were positive for *M. canis*. This suggests that cats without skin lesions could be carriers of this pathogen to humans and other animals. According to this research, humans who are close to and exposed to cats should be more aware of animal diseases, especially skin diseases such as ringworm. Zookeepers, veterinarians, or people who come into contact with these animals should be aware, as contact with infected animals (both at lesions and non-lesion sites) can cause infection. Quick diagnosis leads to rapid treatment, increasing the chance of cats recovering from the disease and reducing the risk of them carrying the pathogen to humans and animals. The current diagnosis method is fungal identification from fungal culture; it takes about 10–14 days for the fungus to fully grow, so the development of a faster diagnostic method would be good for both veterinarians and zookeepers, as well as others who come into contact with animals, as it would reduce the potential risk factors.

Of the 95 cats without skin lesions, ten (10.53%) were positive for *M. canis*. Keepers and those in regular contact with cats should be aware that there is a chance of infection through contact even with healthy cats. There have been reports of *M. canis* causing tinea corporis in humans in Bangkok, Thailand [19]. Two case studies reported symptoms such as redness, dandruff, and scabs, showing severe itching (pruritic erythematous scaly plaques), and found that the symptoms of both cases were caused by *M. canis.* Both patients had a cat in their household. The cats were normal, strong, and healthy cats that did not show any symptoms. Preliminary examinations with Wood’s lamp method provided positive results and diagnoses of *M. canis,* so it is possible that both of these cases were caused by *M. canis* infection from healthy cats to humans. There were no lesions, making humans think that the cats were uninfected and reducing the care taken when dealing with the animal. This increased the risk of infection. Dermatophytosis is a self-curing disease in most animals and also in cats. Infected cats should be isolated from other pets until the disease is clear. The infected cat should be treated because of the zoonosis characteristic of the disease and treatment is usually recommended to shorten the course of the disease and minimize the pathogen to other susceptible animals or humans. Many antifungal drugs have been used successfully for dermatophytosis. Most commonly used antifungal drugs in veterinary medicine were ketoconazole, fluconazole, itraconazole, griseofulvin and terbinafine [20]. Besides the treatment, the prevention is very important; washing hands immediately after contact with animals (with and without skin lesion) every time is necessary when in contact with any animal. The owner should wash/change the bedding frequently because of the contaminated fomite. Mechanical removal of infected organic material/hair and surface washing, with a detergent, is the most important step for environmental cleaning/disinfection. After cleaning, a disinfectant should be used. The veterinarian should educate the owner about the necessity of treatment, control, and prevention of this disease.

## 5. Conclusions

This research focused on the epidemiology of *M. canis* found in cats, collecting samples via the Mackenzie technique from cats with and without lesions of dermatitis. Preliminary examination was done with 10% KOH preparation, followed by culture for fungal identification and confirmation with molecular techniques. Because the pathogen can be found in the hair of cats with and without skin lesions, owners, veterinary staff, and others who come into contact with the animals are at risk of infection if they are not aware or do not take precautions. The zoonotic risk and potential as an etiologic agent for a variety of diseases should be considered and investigated further.

## Figures and Tables

**Table 1:** Number of cats that were positive for *M. canis* by fungal culture and identification.

Cat	Number	Result	Number	Site	Number
With skin lesions	43	+*M. canis*	18	lesion and other site of body	9
				body	2
				lesion	7
		−*M. canis*	25	-	-
Without skin lesions	95	+*M. canis*	10	body	10
		−*M. canis*	85	-	-
Total					28

## Data Availability

Data is contained within the article.
